# Tetracycline Alleviates Cadmium Toxicity in Rice Seedlings by Altering Pollutant Accumulation, Nutrient Absorption, Osmoregulation and Antioxidant Metabolism

**DOI:** 10.3390/molecules30102160

**Published:** 2025-05-14

**Authors:** Ke Li, Yanfang Ren, Xuejie Dong, Xianyi Ping, Junyu He

**Affiliations:** School of Environmental Science and Engineering, Changzhou University, Changzhou 213164, China; xiaolike92@163.com (K.L.); xuejiedong0415@163.com (X.D.); pxy08577@163.com (X.P.)

**Keywords:** tetracycline, cadmium, rice, toxicity

## Abstract

The co-contamination of cadmium (Cd) and tetracycline (TC) in an agricultural environment poses significant risks to plant growth and food safety. This study investigated their combined effects on rice seedlings by analyzing growth parameters, nutrient uptake, photosynthetic pigment levels, antioxidant enzyme activities, nonenzymatic antioxidants, osmoregulatory substances, and secondary metabolites. Results showed that TC alleviated the inhibition of Cd on rice seedling growth by inhibiting Cd accumulation, enhancing nutrient element absorption, facilitating the synthesis of photosynthetic pigments, increasing the activities of antioxidant enzymes and nonenzymatic antioxidants, and the levels of osmoregulatory substances and secondary metabolites. The research provided critical insights into the antagonistic toxicological effects of TC and Cd co-contamination in rice, offering significant information for environmental risk assessments and strategies to alleviate the influence of these pollutants on crop health.

## 1. Introduction

In the last decades, heavy metal pollution has become a global environmental concern affecting sustainable agricultural development and human health. Heavy metals are characterized by high toxicity, insidiousness, persistence, durability, and irreversibility [1]. They disrupt the normal balance of ion absorption, transport, and regulation, thereby interfering with the normal metabolic functions of plants and exerting toxic effects. Heavy metal pollution further poses serious risks to human health through the food chain [2,3]. In addition to heavy metal pollution, the potential threats of antibiotic contamination to the environment and human health cannot be underestimated. With the rapid development of intensive farming, antibiotics have been widely and extensively used in livestock farming, agriculture, and aquaculture [4]. China is the largest global producer and consumer of antibiotics [5]. At present, 44 antibiotics have been detected in soil samples across China, including 4 tetracyclines, 16 quinolones, 15 sulfonamides, and 9 macrolides, with total concentrations ranging from 4.55 to 2010 ng g^−1^ [6]. The application of untreated animal manure and irrigation with wastewater to farmland has been identified as a key potential pathway for antibiotics to enter agricultural ecosystems. Moreover, the long-term accumulation of antibiotics in the environment facilitates the emergence and spread of antibiotic-resistance genes [7]. Antibiotic contamination presents significant threats to both ecosystems and human health [8]. More seriously, in real environmental settings, multiple pollutants often coexist, and their interactions can result in different effects. Recently, the co-contamination of antibiotics and heavy metals has been increasingly prevalent in agro-ecosystems and the potential impacts of such combined pollution on crops have attracted widespread attention [9,10]. Consequently, it is essential to conduct in-depth research on the ecological toxicological mechanisms of heavy metal and antibiotic pollution to better understand their combined environmental and health effects.

Cadmium (Cd), a typical heavy metal pollutant, has been introduced into the environment in large quantities through industrial emissions and the application of chemical fertilizers [11]. Over 0.4 million square kilometers of agricultural land in China experience Cd contamination [12]. Numerous studies have demonstrated that Cd accumulation in soil exhibits high toxicity, inhibiting seed germination of rice, damaging nutrient absorption and redistribution of wheat, suppressing photosynthesis of strawberry, and posing severe threats to crop growth, development, yield, and food safety [13,14,15].

Tetracycline (TC) is characterized by strong adsorption in soil, resistance to degradation, and persistence, especially exhibiting high stability in soil and water systems, compared with other antibiotics [16]. According to previous studies, the accumulation of TC in soil may adversely affect crop growth and physiological functions [17]. It has been found that TC can translocate and accumulate in different parts of the plant tissue and negatively impact the quality and yield of wheat [18]. Li et al. [19] found that TC significantly reduced the biomass of *Chlamydomonas reinhardtii* by inhibiting the generation of photosynthetic pigments. Yagoubi et al. [20] also pointed out that high levels of TC accumulated significantly in the roots and leaves of pea, inhibiting photosynthesis and causing significant oxidative damage, thereby having a negative impact on the biomass of pea. Moreover, TC can influence the structure and function of soil microbial communities, leading to changes in nutrient cycling [20].

The ecological risks posed by combined pollution, including inhibition of growth and functional substances, ROS accumulation, and physiological and metabolic disorders, are more complex compared to single-pollutant scenarios [21]. Antibiotics contain several functional groups such as O-functional groups, which could act as potential electron donors and facilitate complexation with metal ions [9]. The interactions between heavy metals and antibiotics affect their environmental behavior and toxicological effects, resulting in different combined toxicity effects, including synergism, antagonism, or addition [10]. For example, enrofloxacin has been shown to enhance the toxicity of Cd by increasing the Cd accumulation and associated oxidative stress in earthworm [22]. The simultaneous presence of Cd (10 mg kg^−1^) and sulfamethazine (SMT) was observed to influence the accumulation and enrichment of SMT/Cd, leading to a more pronounced impact on pakchoi, which disrupted the antioxidant defense mechanism and eventually detrimentally affects plant growth and quality [23]. Sulfadiazine in conjunction with 50 mg kg^−1^ Cd demonstrated a synergistic impact that inhibited growth and antioxidative enzymatic activity, intensified oxidative stress through ROS production, and increased Cd absorption in the roots and shoots of spinach [24]. The study of Ren et al. [25] illustrated that OPEs mitigated the negative effects of 10 μmol L^−1^ Cd on rice growth. Consequently, examining the toxicity of TC and Cd on various plants’ growth and metabolism is essential for ecological safety and human health [9].

Rice (*Oryza sativa* L.), as a crucial staple crop globally with an annual yield of up to 520 million tons, is adversely impacted by the combined pollution of TC and Cd, which poses potential risks to food security and ecological health. Nonetheless, existing research has predominantly focused on the toxic effects of either TC or Cd alone. Although, He et al. [26] have shown that the interaction between low concentrations of TC and Cd can promote rice root elongation, whereas higher concentrations exhibit the opposite effect. Nevertheless, studies on the toxicity effects and mechanisms of the combined TC and Cd remain highly limited [27], making it challenging to elucidate their interactive mechanisms within rice plants. Based on this, this study systematically examined the effects of TC and Cd co-contamination on the growth and physiological characteristics of rice. This research seeks to enhance our understanding of the ecological toxicological effects of antibiotic and heavy metal co-contamination, providing theoretical insights into the ecological and environmental risks associated with combined pollution.

## 2. Results

### 2.1. Growth Indexes

As shown in Table 1, Cd alone significantly reduced the shoot length, root length, and fresh and dry weights of rice shoots and roots, with the inhibitory effect increasing as the concentration of Cd rose. Similarly, TC also significantly inhibited the growth of rice seedlings. Compared with the control, these growth indexes were reduced by 15.41%, 21.33%, 17.78%, 19.39%, 20.87%, and 33.08%. However, compared to Cd1 treatment, the TC + Cd1 combination significantly (*p* < 0.05) increased the length and fresh and dry weight of both shoots and roots by 11.28%, 9.55%, 13.35%, 12.55%, 19.07%, and 18.75%, respectively. Similarly, in comparison with Cd2, the TC + Cd2 treatment resulted in significant improvements across all these indices.

### 2.2. Cd Content

Under Cd stress, the accumulation of Cd showed a dose effect, which, in the roots, was greater than that in the shoots (Table 2). TF increased by 36.36% with the increase in Cd concentration. Under Cd stress, the co-exposure of TC significantly (*p* < 0.05) decreased the accumulation of Cd in the shoots and roots of rice seedlings by 19.73–26.60% and 10.56–14.10% and also decreased TF by 6.67–18.18%, indicating that TC inhibits the transport of Cd from the roots to the shoots, likely by reducing Cd absorption.

### 2.3. Photosynthetic Pigments

Cd significantly (*p* < 0.05) reduced the contents of chl a, chl b, total chl (chl a + b), and car (Table 3). The chl a, chl b, total chl, and car contents in Cd1 and Cd2 treatments significantly (*p* < 0.05) dropped by 16.22% and 28.65%; 17.52% and 27.40%; 16.15% and 28.18%; and 15.38% and 34.89%, respectively, compared to CK. The chl a and total chl content significantly diminished by 9.73% and 11.00% with TC treatment. Under Cd stress, the co-exposure of TC markedly increased the contents of chl a, chl b, total chl, and car by 14.84–15.15%, 9.09–11.24%, 12.92–13.52%, and 11.36–14.71%, respectively.

### 2.4. Mineral Nutrients

The contents of mineral nutrients in roots and shoots decreased under single TC/Cd treatments (Table 4). Cd treatment significantly (*p* < 0.05) decreased the contents of Ca, Mn, Zn, Cu, and Fe in shoots and roots of rice seedlings by 28.95–43.83% and 29.99–45.09%, 43.16–57.00% and 37.18–49.26%, 18.95–38.59% and 29.83–45.11%, 12.40–25.75% and 35.04–50.24%, and 31.59–50.63% and 30.95–55.58%, respectively, compared with CK. However, the addition of TC significantly increased the contents of Ca, Mn, Zn, Cu, and Fe in the shoots and roots of rice seedlings under Cd1 stress by 9.03% and 14.27%, 14.17% and 23.64%, 10.06% and 26.95%, 5.32% and 22.56%, and 10.81% and 20.60%, respectively, and increased the contents of Ca, Mn, Zn, Cu, and Fe in the shoots and roots under Cd2 stress by 12.58% and 23.43%, 11.16% and 14.83%, 8.63% and 11.51%, 8.19% and 13.16%, and 15.73% and 25.05%.

### 2.5. ROS

**Figure 1:**
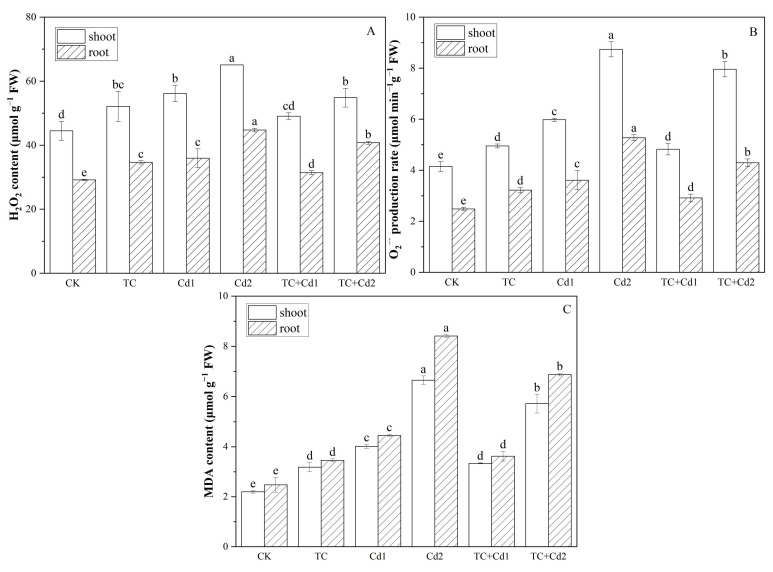
Impacts of TC/Cd single/combined treatments on H_2_O_2_ (**A**), O_2_^−·^ (**B**), and MDA (**C**) contents of rice seedlings. Different lowercase letters indicate statistically significant difference among treatments at *p* < 0.05.

**Figure 2:**
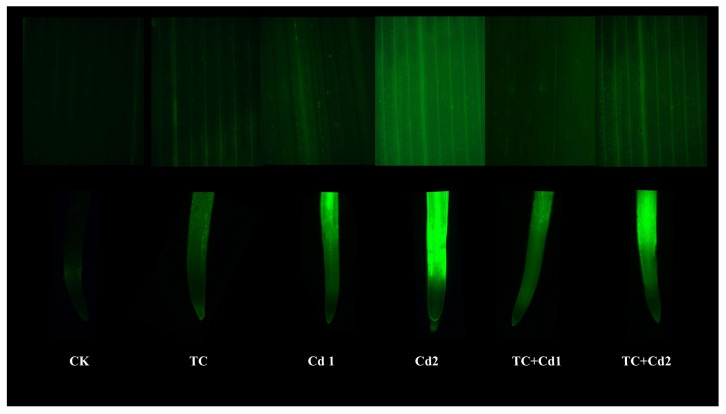
ROS fluorescence staining of root tips and leaves of rice seedlings under single and combined TC/Cd stress.

Under single TC/Cd treatment, the addition and increase in TC and Cd significantly (*p* < 0.05) increased the production rate of O_2_^−·^ and the contents of H_2_O_2_ and MDA in rice seedlings (Figure 1). Compared with Cd1, the co-exposure of TC significantly decreased the contents of H_2_O_2_ and MDA and the production rate of O_2_^−·^ in the shoots and roots of rice seedlings by 12.62% and 12.70%, 16.85 and 18.60%, and 19.31% and 19.28%, respectively. The addition of TC also reduced the contents of H_2_O_2_ and MDA and the production rate of O_2_^−·^ under Cd2 treatment. It can also be seen from the ROS fluorescence staining diagram that the addition of TC reduced the fluorescence intensity of rice seedlings under Cd stress (Figure 2).

### 2.6. Antioxidant Enzymes

**Figure 3:**
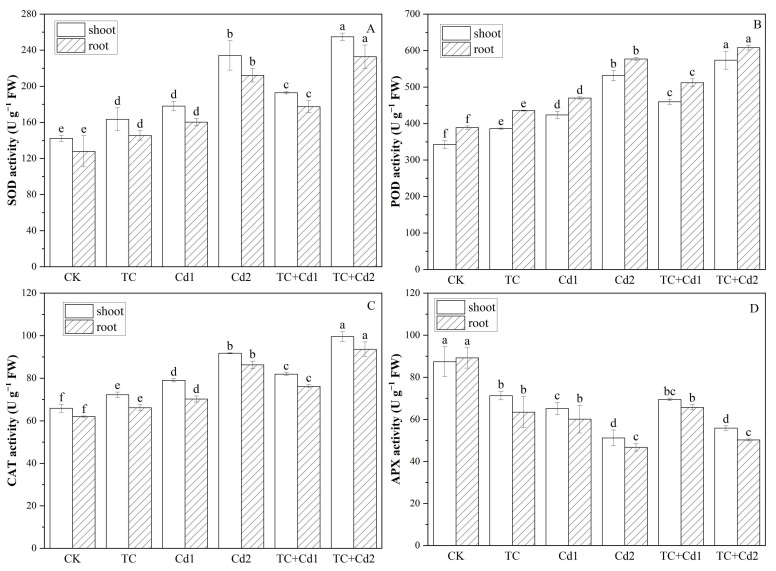
Impacts of TC/Cd single/combined treatments on antioxidant enzymes’ (SOD (**A**), POD (**B**), CAT (**C**), and APX (**D**)) activity of rice seedlings. Different lowercase letters indicate statistically significant difference among treatments at *p* < 0.05.

Both single treatments with TC and Cd significantly (*p* < 0.05) increased the activities of SOD, POD, and CAT while reducing the activity of APX in rice seedlings when compared to CK (Figure 3). Under Cd1 stress, the activities of SOD, POD, and CAT in shoots and roots increased by 25.06% and 25.33%, 23.55% and 20.81%, and 19.89% and 13.32%, respectively; the activity of APX dropped by 25.45% and 32.61%, respectively, compared to CK. Similarly, under Cd2 stress, these enzymes’ activities increased by 64.49% and 65.99%, 54.96% and 48.36%, and 39.19% and 39.24%, respectively, relative to CK. The addition of TC significantly rose the activities of SOD (8.35% and 10.72%), POD (7.85% and 8.94%), CAT (3.70% and 8.50%), and APX (9.14% and 7.37%) in both shoots and roots of rice seedlings under Cd1 treatment. Moreover, under Cd2 stress, the co-exposure of TC lessened the activities of SOD (2.30% and 18.7%), POD (14.15% and 15.77%), and CAT (7.11% and 10.43%). However, TC had no significant impact on APX activity under Cd stress.

### 2.7. Osmotic Regulatory Substances

**Figure 4:**
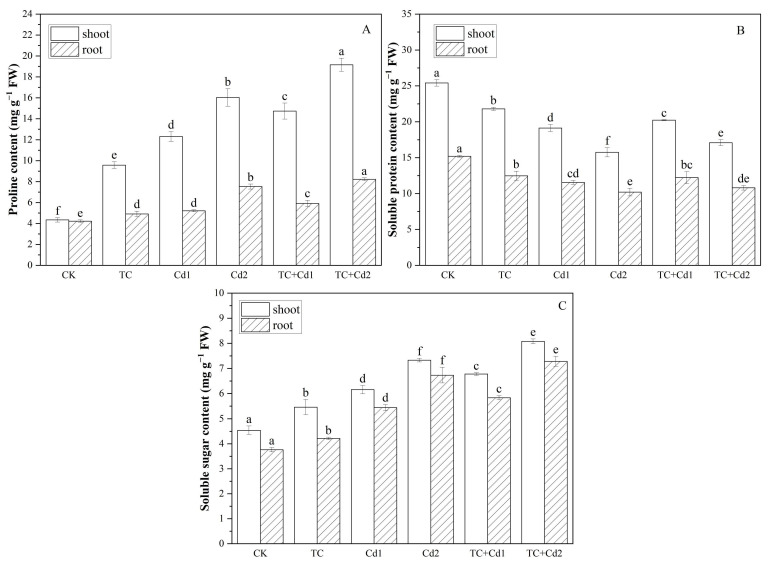
Impacts of TC/Cd single/combined treatments on osmotic regulatory substances (proline (**A**), soluble protein (**B**), and soluble sugar (**C**)) contents of rice seedlings. Different lowercase letters indicate significant differences among different treatments at *p* < 0.05.

All treatments with TC and Cd significantly increased the contents of proline and soluble sugar and lessened the contents of soluble protein in rice seedlings (Figure 4). Notably, the addition of TC resulted in an increase in the proline content (19.49–19.75%), soluble protein content (5.70–8.44%), and soluble sugar content (10.08–10.19%) in the shoots of rice seedlings and 9.14–13.50% proline content and 7.30–8.16% soluble sugar in the roots of rice seedlings under Cd stress.

### 2.8. Secondary Metabolism

**Figure 5:**
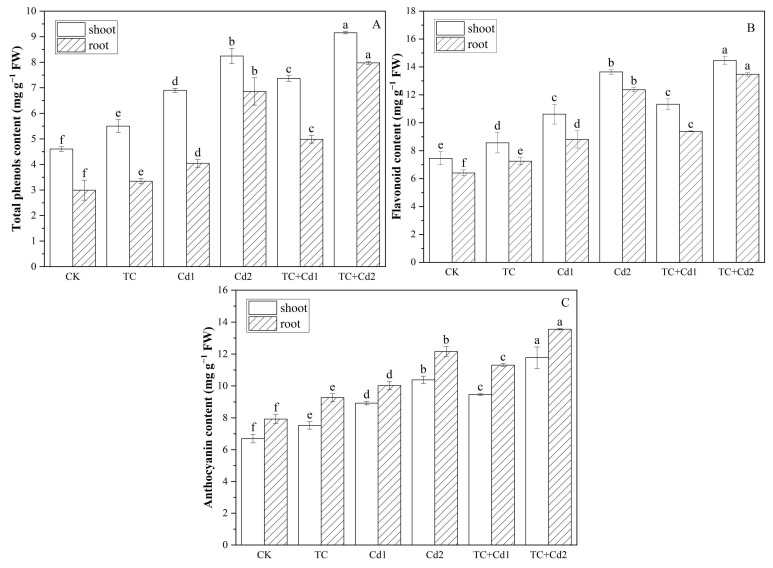
Impacts of TC/Cd single/combined treatments on secondary metabolism (total phenols (**A**), flavonoid (**B**) and anthocyanin (**C**)) contents of rice seedlings. Different lowercase letters indicate significant difference among different treatments at *p* < 0.05.

Both single TC and Cd treatments significantly (*p* < 0.05) increased the contents of total phenols, flavonoids, and anthocyanins in the shoots and roots of rice seedlings, compared with CK, exhibiting a dose-dependent effect (Figure 5). The addition of TC significantly increased the contents of total phenols, flavonoids, and anthocyanins in the shoots and roots of rice seedlings under Cd stress by 6.70–11.05% and 16.33–23.36%, 6.03–6.69% and 6.57–8.94%, and 6.04–13.33% and 11.48% and 12.78%, respectively.

### 2.9. AsA and GSH

**Figure 6:**
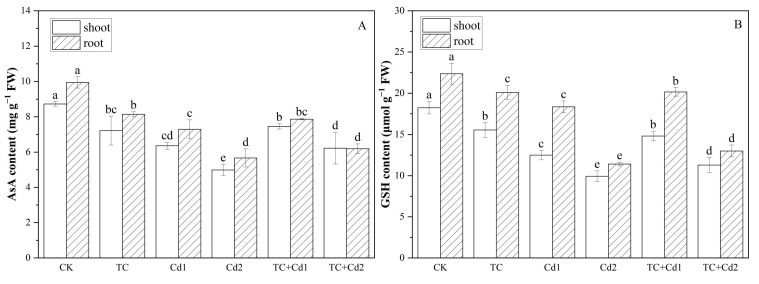
Impacts of TC/Cd single/combined treatments on AsA (**A**) and GSH (**B**) contents of rice seedlings. Different lowercase letters indicate significant differences among different treatments at *p* < 0.05.

Under single TC/Cd stress, the contents of AsA and GSH in rice seedlings decreased significantly (*p* < 0.05) with the increase in TC/Cd concentration (Figure 6). The addition of TC increased the AsA and GSH contents in rice seedlings subjected to Cd stress. In comparison to Cd1 and Cd2, the co-exposure to TC increased the contents of AsA and GSH by 17.26% and 18.53% and 24.82% and 13.67% in shoots, as well as 7.85% and 9.58% and 9.30% and 13.06% in roots, respectively.

### 2.10. Principal Component Analysis (PCA) and Heat Map

**Figure 7:**
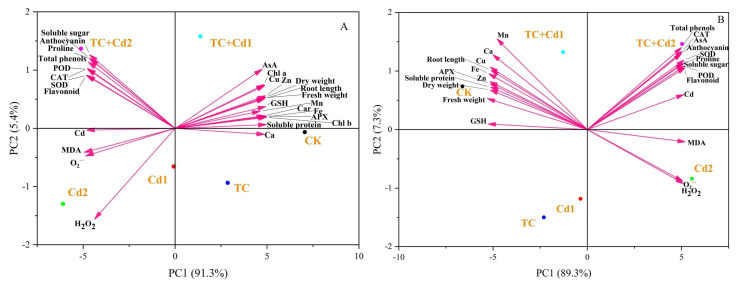
PCA analysis under different treatments: comparison of shoots (**A**) and roots (**B**) of rice seedlings.

The differences in growth and stress responses between CK and different experimental treatments were highlighted by PCA (Figure 7). Component 1 (PC1) and Component 2 (PC2) accounted for 91.3% and 5.4% and 89.3% and 7.3% of the total variability in shoots and roots, respectively. The growth parameters (length and fresh and dry weight), APX activity, GSH, nutritional elements, photosynthetic pigments, and soluble protein in shoots exhibited a positive correlation with PC1. Cd, SOD, POD, CAT, O_2_^−·^, H_2_O_2_, and MDA were negatively correlated with PC1. All treatments except TC showed significant separation from CK, indicating that exposure to Cd single and combined stress significantly affected plant growth and physiology. In both shoots and roots, TC + Cd1 and TC + Cd2 were distributed in the opposite direction of the *y*-axis compared with Cd1 and Cd2, which means that the combination treatments (TC + Cd) produce different biological effects or responses compared to the individual Cd treatments. This contrast suggested potential interaction effects between TC and Cd, which may mitigate the toxicity of Cd on the organism.

**Figure 8:**
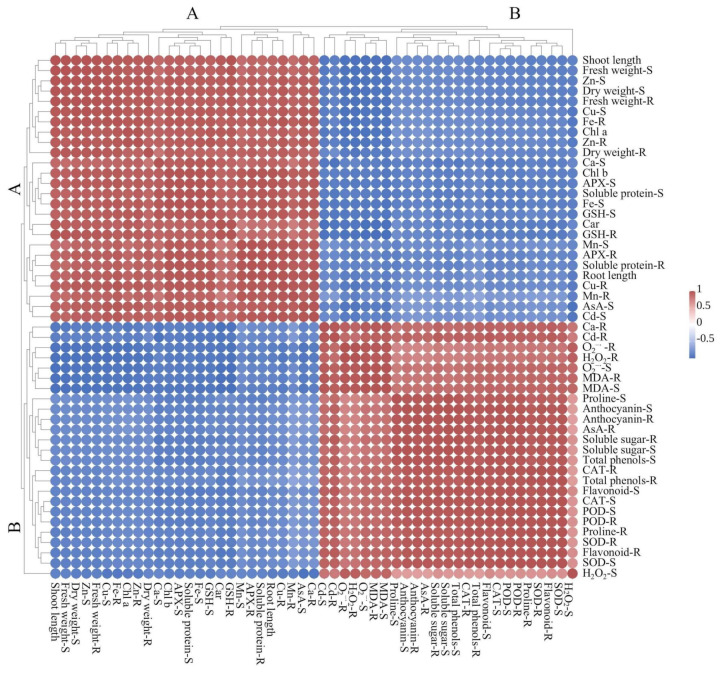
The heat map analysis of the Pearson correlation coefficient among several parameters. S: shoot; R: root. The blue and red in the matrix boxes signified robust negative and positive correlations.

Hierarchical cluster analysis divides all measured parameters into two clusters (A and B); growth parameters, APX activity, nutrient elements, photosynthetic pigments, soluble protein, and nonenzymatic antioxidants (AsA and GSH) were classified as cluster A and biochemical stress-related markers (O_2_^−·^, H_2_O_2_, and MDA), proline, soluble sugar, SOD, POD, CAT, AsA, and secondary metabolites were classified as cluster B (Figure 8). Growth parameters (length and fresh and dry weight) were strongly positively correlated with nutrient elements, photosynthetic pigments, soluble protein, osmoregulatory substances, and nonenzymatic antioxidants. Biochemical stress-related markers (O_2_^−·^, H_2_O_2_, and MDA) showed negative correlations with growth-related variables, highlighting the trade-off between stress response and growth. The positive correlations among antioxidant enzymes (SOD, POD, and CAT), secondary metabolites, osmoregulatory substances (proline and soluble sugar), nonenzymatic antioxidants (AsA), and biochemical stress-related markers suggested that rice plants co-ordinated a range of physiological and biochemical mechanisms to balance growth and stress responses. This is achieved through the synergistic regulation of antioxidant systems, secondary metabolites, and osmoregulatory substances. These components collectively mitigate oxidative stress and minimize cellular damage, thereby enhancing the plant’s ability to withstand stress and maintain physiological stability under adverse conditions.

## 3. Discussion

Plant growth serves as a primary indicator for evaluating the toxicity of pollutants. Single TC and Cd significantly inhibited the growth of rice seedlings, as evidenced by reduced plant height, root length, and fresh and dry weight (Table 1). Additionally, a comparable inhibitory effect of TC on plant biomass was discovered, with 10 mg L^−1^ TC reducing the biomass of *M. aquaticum* by 17% [28]. Furthermore, with increasing stress concentrations, the growth parameters of the rice seedlings generally exhibited a downward trend, which may be because more Cd was accumulated in rice seedlings with the increase in Cd concentration (Table 2). The addition of TC increased plant height, root length, and both fresh and dry weight, hence promoting the growth of rice seedlings under Cd stress (Table 1), indicating that TC may alleviate the inhibitory effects of Cd stress on the growth of rice seedlings. This may be attributed to the potential role of TC in reducing the accumulation of Cd in both shoots and roots of rice seedlings, as well as the translocation factor (Table 2). The addition of TC may restrict Cd accumulation and translocation by competing with Cd for binding sites on the root surface, resulting in increased Cd accumulation in the root system and reduced accumulation in the aboveground parts [26]. Feng et al. [29] reported analogous findings that the addition of oxytetracycline (OTC) increased the accumulation of arsenic (III) in the roots of *Medicago sativa* seedlings.

Nutrients are crucial for plant growth because they not only serve as the basic components required for plant life processes, but also participate in vital activities [30]. This study found that Cd significantly decreased the contents of essential mineral elements, including Ca, Mn, Cu, Fe, and Zn, in the shoots and roots of rice seedlings (Table 4). This may be because the presence of Cd disrupted the function of root nutrient transporters, damaged cell membranes, and even competed with essential minerals for uptake through similar transport channels [31]. In comparison to the single Cd treatment, the coexistence of TC increased the overall content of all mineral nutrients (Table 4). This may be because TC alters transporter activity or improves root metabolism [32], thereby alleviating the negative effects of Cd on trace element absorption to some extent. Li et al. [33] also reported that nanoplastics promoted the absorption of Ca, Fe, and Cu by dandelion under Cd stress. The deficiency of nutrients directly affects the synthesis of photosynthetic pigments [34]. As shown in Table 3, Cd stress decreased the content of photosynthetic pigments in rice seedlings to varying degrees. Under TC combined stress, the contents of photosynthetic pigments were higher compared to the Cd treatments, indicating that TC and Cd exhibited an antagonistic effect on pigment synthesis. Higher contents of photosynthetic pigments are conducive to plant growth, resulting in less toxicity. Similarly, Zong et al. [35] also reported that the presence of polystyrene nanoplastics reduced the accumulation of cadmium in wheat seedlings, resulting in an increase in chlorophyll content.

Pollution stress in the environment often greatly increases the production of ROS (H_2_O_2_ and O_2_^−·^), resulting in oxidative stress [36]. The cytoplasmic membrane is the most vulnerable component of the cell and also the site where damage occurs first when plants are subjected to environmental stress [30]. Under stress, ROS accumulates in plants, resulting in damage to membrane lipids and proteins and the stability of membrane structure and its function are damaged [37]. MDA is a primary byproduct of lipid peroxidation induced by reactive oxygen species (ROS) [30]. In this study, Cd stress caused obvious ROS accumulation and oxidative damage in rice seedlings; however, TC decreased the accumulation of ROS in rice seedlings under Cd stress (Figure 1 and Figure 2). This result was similar to the study conducted by Qiao et al. [38], where both single decabromodiphenyl ethane (DBDPE) and Cd increased ROS and MDA accumulation in lettuce (*Lactuca sativa* L.), while DBDPE decreased the accumulation of ROS and MDA content during Cd stress by up-regulating the activities of SOD, POD, and CAT, hence reducing Cd toxicity.

Plants have evolved many defensive mechanisms to alleviate damage caused by Cd and TC stress [30]. Antioxidant enzymes such as SOD, CAT, POD, and APX are important protective mechanisms of plants [1]. This study showed that SOD, POD, and CAT activities of rice seedlings were significantly increased under single Cd stress (Figure 3), while APX showed an opposite tendency, which may be related to the Cd accumulation and overproduction of ROS force plants to maintain a balance between free radical production and scavenging by increasing antioxidant enzyme activity. However, the drop in APX may be because Cd inhibits the production of AsA and GSH [34], both of which are important nonenzymatic compounds involved in oxidative stress and serve as substrates for the production of antioxidant enzymes. This conclusion is consistent with our research findings, which show that Cd inhibited the production of AsA and GSH in rice seedlings (Figure 6), and that APX activity decreased. Nevertheless, the addition of TC resulted in a substantial increase in the contents of AsA and GSH and the activity of all antioxidant enzymes. These results indicated that TC addition stimulated the generation of antioxidants in rice seedlings and, thus, weakened the oxidative damage of Cd on seedlings. Ben et al. [39] also reported that low concentrations of lanthanum increased the activity of antioxidant enzymes by facilitating the absorption of trace elements, so ultimately reducing the cytotoxicity of lead.

Substances in plant cells such as soluble sugars, soluble proteins, proline, etc., can stabilize the intracellular water balance by regulating the osmotic potential of cells, removing the generated free radicals, protecting the cell membrane structure, and keeping it intact [40]. In this study, it was found that, under single Cd stress, the contents of proline and soluble sugar in rice seedlings were significantly increased but the contents of soluble protein decreased in comparison with CK. The increase in proline may be because TC/Cd stress inhibited the activity of proline degrading enzyme (PRODH) and promoted the glutamate synthesis pathway of proline [41]. Research has shown that rice can promote the accumulation of soluble sugars and maintain osmotic equilibrium in cells by regulating the expression of soluble-sugar-related genes in starch and sucrose metabolic pathways [42]. Conversely, TC/Cd inhibits the growth of rice seedlings, resulting in a decrease in their utilization of soluble sugar. Thus, the accumulation of soluble sugar in rice seedlings was promoted [43]. TC significantly increased the contents of proline, soluble protein, and soluble sugar in rice seedlings under Cd stress (Figure 4), indicating that TC protected cellular structure and maintained metabolic function under Cd stress [33].

Total phenols, flavonoids, and anthocyanins are essential secondary metabolites in plants, acting as antioxidants to mitigate plant stress [44]. This investigation has shown that single Cd treatments significantly decreased the contents of these compounds in both shoots and roots (Figure 5), but TC increased total phenols, flavonoids, and anthocyanins in rice seedlings under Cd stress, indicating an enhancement of secondary metabolic reaction. The notable alterations in phenolic compounds under TC/Cd stress may be attributed to the modulation of enzymes like phenylalanine ammonia-lyase (PAL), chalcone synthase (CHS), and other associated enzymes [45]. Following the TC + Cd treatment, rice seedlings exhibited an increased accumulation of proline (Figure 5), which subsequently enhanced the activity of the oxidative pentose phosphate pathway (PPP). This pathway is crucial for providing necessary precursors for the biosynthesis of phenolic compounds [46]. The results indicated that complex interactions between TC and Cd can regulate metabolic pathways for secondary metabolite biosynthesis [47,48].

## 4. Materials and Methods

### 4.1. Plant Materials and Reagents

Rice seeds (*Oryza sativa* L. cv. Zhongyou 169) were purchased from Quanyin High Technology Seed Co., Ltd. (Hefei, China). TC (≥98.0%) was purchased from Aladdin Reagents Ltd. (Shanghai, China). CdCl_2_ (99.99%) was purchased from Shanghai RichJoint Chemical Reagents Co., Ltd. (Shanghai, China).

### 4.2. Plant Culture and Treatments

Rice seedlings were cultured using the procedure described by Gu et al. [1]. Uniform and healthy seedlings at 12 days old were transplanted into hydroponic containers (covered with aluminum foil) with a complete Hoagland’s nutritional solution and subjected to the following treatments: (1) control (CK); (2) 4.50 mg L^−1^ TC (TC); (3) 1.18 mg L^−1^ Cd (Cd1); (4) 5.80 mg L^−1^ Cd (Cd2); (5) 4.50 mg L^−1^ TC + 1.18 mg L^−1^ Cd (TC + Cd1); and (6) 4.50 mg L^−1^ TC + 5.80 mg L^−1^ Cd (TC + Cd2). Each treatment was duplicated three times. The concentrations of Cd1 and Cd2 were selected according to our preliminary experiments conducted by Jia et al. [49], which inhibited the rice seedling biomass by about 25% and 50%, respectively. The TC concentration was selected guided by the values from the literature on relevant environmental matrices. TC concentration in cattle and pig manure ranges from trace levels (≤0.1 mg kg^−1^) to a maximum of 66 mg kg^−1^, while, in soil improved with manure, TC concentration is usually one to three orders of magnitude lower [50]. He et al. [26] found TC over 10 μmol L^−1^ significantly inhibited rice seedling growth. The seedlings were treated for 12 d, with the nutrient solution replaced every 3 d. After 12 d of treatment, one part of the samples was collected for measuring growth index and photosynthetic pigment contents, and the other part was stored at −80 °C.

### 4.3. Determination of Rice Growth Indexes

Harvested rice plants were thoroughly rinsed with distilled water. The shoot length and root length of rice seedlings were measured using Image-Pro Plus 6.0 software. The rice plants were then divided into shoots and roots, and the fresh weight (FW) of each part was measured using an analytical balance (Ohaus, PR224ZH, Shanghai, China). Subsequently, the samples were dried at 80 °C until a constant weight was achieved, and the dry weight (DW) of each part was measured.

### 4.4. Determination of the Contents of Cd, Nutrient Elements, and Transport Factor

Dried rice sample powder (0.25 g) was digested with 7 mL 80% nitric acid on the digital hot plate (Uchen, LC-DB-2EFS, Beijing, China). The contents of Cd and nutrient elements (Ca, Mn, Zn, Fe, and Mg) were analyzed by flame atomic absorption spectrophotometer (AAS, aa-300, Perkin Elmer Ltd., Shelton, CT, USA). The transport factors (TF) of Cd represented the ratio of the content of Cd in shoot to that of in root.

### 4.5. Photosynthetic Pigments

Leaf sample (0.2 g) was extracted with 80% acetone and the absorbance of the extract was measured at 663 nm, 645 nm, and 470 nm, respectively. The contents of chlorophyll a/b (chl a/b), total chlorophyll (chl a + b), and carotenoids (car) were calculated according to the method of Ren et al. [34] and expressed as mg g^−1^ FW.

### 4.6. Reactive Oxygen Species (ROS), Malondialdehyde (MDA) Contents, and Histochemical Detection

The contents of hydrogen peroxide (H_2_O_2_) and the superoxide anion (O_2_^−·^) production rate were measured and calculated in accordance with the methodology described by Jia et al. [49]. For the determination of H_2_O_2_ level, the extraction of 3 mL was mixed with 1 mL of 0.1% TiCl_4_ in 20% H_2_SO_4_ (*v*/*v*), and the mixture was then centrifuged at 6000× *g* for 15 min. The peroxide–titanium complex was dissolved in 3 mL 1 mmol L^−1^ H_2_SO_4_. The absorbance was measured at 410 nm. For the determination of O_2_^−·^ production rate, an aliquot of the extraction was added to incubation buffer consisting of 50 mmol L^−1^ potassium phosphate buffer (pH 7.8) and 1 mmol L^−1^ hydroxylamine hydrochloride, incubating the mixture at 25 °C for 20 min. Then, it was added to 17 mmol L^−1^ aminobenzene sulphonic acid and 7 mmol L^−1^ α-naphthylamine and incubated again at 25 °C for 20 min. Absorbance was measured at 530 nm. The H_2_O_2_ content was expressed as μmol g^−1^ FW and O_2_^−·^ production rate was expressed as μmol g^−1^ FW min^−1^. MDA content was measured by thiobarbituric acid methodology described by Wang et al. [36]. A total of 2.0 mL of the extraction was added to 4.0 mL of thiobarbituric acid (TBA, 0.67%) with 20% trichloroacetic acid (TCA), heated at 100 °C for 30 min, and then quickly cooled to room temperature. Absorbance of the supernatant was measured at 450 nm, 532 nm, and 600 nm, respectively, after centrifugation at 10,000× *g* for 10 min. MDA contents were expressed as μmol g^−1^ FW.

The generation of ROS in the samples was evaluated using 2′,7′-dichlorodihydrofluorescein diacetate (DCFH-DA) staining. Rice leaves or roots were incubated with DCFH-DA (10 μmol L^−1^) for 40 min at 20 °C in the darkness. The tissues were observed and captured using a fluorescent microscope (Mingmei, MF53-N, Guangzhou, China).

### 4.7. Antioxidant Enzyme Activity

Fresh samples (0.1 g) were homogenized in 1 mL 50 mmol L^−1^ potassium phosphate buffer (pH 7.8) containing 1% polyvinylpyrrolidone and 1.33 mmol L^−1^ EDTA using a chilled pestle and mortar. The homogenate was centrifuged at 12,000× *g* for 20 min at 4 °C, and the supernatant was collected for the analyses of superoxide dismutase (SOD), peroxidase (POD), catalase (CAT), and ascorbate peroxidase (APX) activities as previously described by Ji et al. [30]. One unit of SOD activity was defined as the amount of enzyme that would inhibit 50% of NBT photoreduction at 560 nm. One-unit POD, CAT, and APX activities were defined as an absorbance change of 0.1, 0.1, and 0.1 units in 1 min at 470 nm, 240 nm, and 290 nm, respectively.

### 4.8. Determination of Osmolytes

Soluble sugar was measured by anthrone colorimetric method [40]. A total of 0.2 g sample was extracted with 4 mL 80% methanol at 80 °C for 40 min. After centrifugation, the mixture containing 4 mL of anthrone and the supernatant was heated in a boiling water bath for 10 min and then cooled. Absorbance was measured at 620 nm. Soluble protein content was determined at 595 nm by the Coomassie brilliant blue method, and bovine serum protein was used as the standard curve. The content of proline was determined by the acid ninhydrin method [51]. A total of 0.2 g sample was homogenized with 5 mL 3% sulphosalicylic acid solution. After centrifugation, the supernatant (2 mL) was combined with 2 mL of glacial acetic acid, 2 mL of acid ninhydrin, and 2 mL of water, boiled at 100 °C for 1 h, and then cooled. The mixture was then extracted with 4 mL of toluene and thoroughly mixed. The free toluene was quantified at 520 nm. The contents were shown as mg g^−1^ FW.

### 4.9. Determination of Secondary Metabolites

Total phenol, flavonoid, and anthocyanin contents were measured in accordance with the approach outlined by Gu et al. [1]. The absorbance of the reaction mixture was measured at 750 nm, 510 nm, and 530 nm, respectively, with the results expressed as mg g^−1^ FW.

### 4.10. Determination of Ascorbic Acid (AsA) and Glutathione (GSH)

AsA and GSH were determined using the method previously described by Kaya et al. [52]. For AsA extraction, fresh sample (0.2 g) was homogenized on ice with 1 mL of 5% (*w*/*v*) TCA and centrifuged at 10,000× *g* for 10 min. The reaction mixture included 2.0 mL of 0.1 mol L^−1^ phosphate buffer (pH 6.0), 100 μL supernatant, and 500 μL distilled water. Absorbance of the reaction solution was measured at 265 nm, and AsA content was expressed as mg g^−1^ FW. For GSH extraction, fresh sample (0.2 g) was homogenized with 2 mL of 5% (*w*/*v*) potassium–phosphoric acid in 1 mmol L^−1^ ethylenediaminetetraacetic acid (EDTA) and centrifuged at 14,000× *g* for 10 min. Absorbance of the reaction solution containing the supernatant and 4 mmol L^−1^ 5,5′-dithiobis (2-nitrobenzoic acid) (DTNB) was measured at 412 nm. GSH content was expressed as μmol L^−1^ FW.

### 4.11. Statistical Analysis

All data are shown as the average of three replicates ± standard deviation (SD). The data were analyzed using one-way analysis of variance (ANOVA) by SPSS 26.0 (SPSS, Inc., Chicago, IL, USA). Duncan’s multiple-range test was employed to assess significant differences across the treatments (*p* < 0.05). Principal component analysis (PCA) and correlation analysis using the Pearson correlation coefficient were utilized to examine and illustrate the interdependence across indices.

## 5. Conclusions

This study revealed that 4.50 mg L^−1^ TC enhanced the defense mechanisms of rice seedlings under Cd stress by optimizing the interactions among antioxidants, osmoregulatory chemicals, and secondary metabolites. Specifically, TC reduced Cd uptake by restricting its translocation from roots to shoots, which led to lower Cd accumulation and mitigated its toxic effects. Furthermore, TC promoted the absorption of essential nutrients and facilitated the formation of photosynthetic pigments in the seedlings, therefore promoting plant growth and alleviating Cd-induced toxicity. This research provides valuable insights into the antagonistic relationship between TC and Cd in agricultural environments. This study suggests that application of TC may be possible to mitigate Cd accumulation in crops, which has significant implications for reducing the risks of Cd contamination in the food chain. Further investigation is required to elucidate the molecular mechanisms through which TC alleviates Cd toxicity for developing more effective strategies for managing co-contamination in agricultural systems.

## Figures and Tables

**Table 1:** Growth indexes of rice seedlings under different treatments. Different lowercase letters indicate statistically significant difference among treatments at *p* < 0.05.

Treatments	Plant Height (cm)	Root Length (cm)	Fresh Weight (mg Plant^−1^)		Dry Weight (mg Plant^−1^)	
			Shoot	Root	Shoot	Root
CK	54.25 ± 2.20 ^a^	23.54 ± 0.79 ^a^	1128.46 ± 92.30 ^a^	1170.53 ± 60.46 ^a^	172.93 ± 10.54 ^a^	78.75 ± 4.71 ^a^
TC	45.89 ± 1.23 ^c^	18.52 ± 0.12 ^b^	927.81 ± 20.69 ^c^	943.57 ± 35.94 ^c^	136.84 ± 1.80 ^c^	52.70 ± 1.51 ^c^
Cd1	44.05 ± 3.55 ^c^	17.18 ± 0.48 ^c^	925.52 ± 9.99 ^c^	929.71 ± 15.44 ^c^	130.51 ± 2.25 ^c^	59.00 ± 1.28 ^d^
Cd2	28.81 ± 0.68 ^e^	14.41 ± 0.34 ^e^	588.12 ± 21.51 ^e^	639.45 ± 9.33 ^e^	87.29 ± 1.99 ^e^	32.99 ± 0.70 ^f^
TC + Cd1	49.02 ± 0.59 ^b^	18.82 ± 0.52 ^b^	1009.04 ± 18.03 ^b^	1016.42 ± 2.63 ^b^	149.40 ± 3.05 ^b^	64.06 ± 3.07 ^b^
TC + Cd2	32.37 ± 1.08 ^d^	16.07 ± 0.61 ^d^	666.36 ± 5.98 ^d^	703.06 ± 1.40 ^d^	97.15 ± 3.22 ^d^	36.98 ± 0.67 ^e^

**Table 2:** Accumulation and transport factor (TF) of Cd in rice seedlings under different treatments. Different lowercase letters indicate statistically significant difference among treatments at *p* < 0.05.

Treatments	Cd Content (mg kg^−1^)		TF
	Shoot	Root	
Cd1	71.95 ± 1.84 ^c^	667.74 ± 14.28 ^c^	0.11
Cd2	208.25 ± 4.83 ^a^	1350.73 ± 23.94 ^a^	0.15
TC + Cd1	52.81 ± 2.10 ^d^	573.62 ± 13.58 ^d^	0.09
TC + Cd2	167.17 ± 1.29 ^b^	1208.03 ± 26.67 ^b^	0.14

**Table 3:** Photosynthetic pigment content of rice seedlings under different treatments. Different lowercase letters indicate statistically significant difference among treatments at *p* < 0.05.

Treatments	Chl a (mg g^−1^)	Chl b (mg g^−1^)	Total Chl (mg g^−1^)	Car (mg g^−1^)
CK	1.85 ± 0.01 ^a^	1.06 ± 0.03 ^a^	2.91 ± 0.03 ^a^	0.52 ± 0.02 ^a^
TC	1.74 ± 0.03 ^b^	0.92 ± 0.04 ^ab^	2.66 ± 1.01 ^b^	0.51 ± 0.01 ^ab^
Cd1	1.61 ± 0.02 ^c^	0.87 ± 0.01 ^ab^	2.48 ± 0.02 ^c^	0.45 ± 0.02 ^c^
Cd2	1.44 ± 0.09 ^e^	0.77 ± 0.23 ^b^	2.21 ± 2.01 ^e^	0.34 ± 0.01 ^e^
TC1 + Cd1	1.78 ± 0.02 ^b^	0.91 ± 0.01 ^ab^	2.69 ± 0.03 ^b^	0.49 ± 0.02 ^b^
TC1 + Cd2	1.52 ± 0.02 ^d^	0.80 ± 0.02 ^b^	2.32 ± 0.32 ^d^	0.36 ± 0.01 ^d^

**Table 4:** Impacts of TC/Cd single and combined treatments on mineral nutrient contents of shoots and roots of rice seedlings. Different lowercase letters indicate statistically significant difference among treatments at *p* < 0.05.

Treatments		Ca (mg kg^−1^)	Mn (mg kg^−1^)	Zn (mg kg^−1^)	Cu (mg kg^−1^)	Fe (mg kg^−1^)
CK	Shoot	454.65 ± 5.00 ^a^	187.34 ± 7.58 ^a^	147.18 ± 6.54 ^a^	29.57 ± 1.29 ^a^	211.25 ± 12.19 ^a^
	Root	128.60 ± 9.80 ^a^	299.01 ± 5.14 ^a^	107.63 ± 4.78 ^a^	36.27 ± 1.13 ^a^	331.59 ± 12.61 ^a^
TC	Shoot	420.63 ± 7.62 ^b^	115.67 ± 3.23 ^b^	123.65 ± 4.37 ^c^	26.43 ± 0.69 ^b^	172.46 ± 6.34 ^b^
	Root	99.42 ± 2.68 ^b^	193.64 ± 6.25 ^c^	91.08 ± 4.55 ^b^	26.38 ± 1.10 ^c^	249.83 ± 4.67 ^c^
Cd1	Shoot	323.05 ± 5.18 ^d^	106.48 ± 7.04 ^c^	119.28 ± 3.21 ^c^	25.90 ± 1.39 ^b^	144.51 ± 7.12 ^d^
	Root	90.03 ± 3.03 ^c^	187.83 ± 8.32 ^c^	75.53 ± 1.72 ^c^	23.56 ± 0.64 ^d^	228.96 ± 13.90 ^d^
Cd2	Shoot	255.36 ± 9.43 ^f^	80.55 ± 1.33 ^e^	90.39 ± 1.28 ^e^	21.95 ± 1.56 ^d^	104.29 ± 6.75 ^f^
	Root	70.62 ± 0.99 ^d^	151.73 ± 2.49 ^e^	59.08 ± 8.63 ^e^	18.05 ± 1.05 ^f^	147.30 ± 6.17 ^f^
TC + Cd1	Shoot	352.22 ± 5.36 ^c^	121.57 ± 1.21 ^b^	131.29 ± 1.86 ^b^	27.28 ± 0.39 ^b^	160.13 ± 2.16 ^c^
	Root	102.87 ± 2.63 ^b^	232.24 ± 2.57 ^b^	95.88 ± 2.75 ^b^	28.88 ± 0.15 ^b^	276.13 ± 2.90 ^b^
TC + Cd2	Shoot	287.48 ± 2.41 ^e^	89.54 ± 1.17 ^d^	98.19 ± 2.38 ^d^	22.37 ± 0.17 ^c^	120.69 ± 7.56 ^e^
	Root	87.16 ± 4.09 ^c^	174.23 ± 1.29 ^d^	65.88 ± 4.68 ^d^	20.43 ± 0.30 ^e^	184.20 ± 2.69 ^e^

## Data Availability

The original contributions presented in the study are included in the Article, further inquiries can be directed at the corresponding authors.
